# Improving the Detection Accuracy of Subsurface Damage in Optical Materials by Exploiting the Fluorescence Polarization Properties of Quantum Dots

**DOI:** 10.3390/nano15151182

**Published:** 2025-07-31

**Authors:** Yana Cui, Xuelian Liu, Bo Xiao, Yajie Wu, Chunyang Wang

**Affiliations:** 1 School of Electronic and Information Engineering, Ningbo University of Technology, Ningbo 315211, China; 2 Xi’an Key Laboratory of Active Photoelectric Imaging Detection Technology, Xi’an Technological University, Xi’an 710021, China

**Keywords:** subsurface damage detection, quantum dots, optical material, fluorescence polarization

## Abstract

Optical materials are widely used in large optical systems such as lithography machines and astronomical telescopes. However, optical materials inevitably produce subsurface damage (SSD) during lapping and polishing processes, degrading the laser damage threshold and impacting the service life of the optical system. The large surface roughness of the lapped optical materials further increases the difficulty of the nondestructive detection of SSD. Quantum dots (QDs) show great development potential in the nondestructive detection of SSD in lapped materials. However, existing QD-based SSD detection methods ignore the polarization sensitivity of QDs to excitation light, which affects the detection accuracy of SSD. To address this problem, this paper explores the fluorescence polarization properties of QDs in the SSD of optical materials. First, the detection principle of SSD based on the fluorescence polarization of QDs is investigated. Subsequently, a fluorescence polarization detection system is developed to analyze the fluorescence polarization properties of QDs in SSD. Finally, the SSD is detected based on the studied polarization properties. The results show that the proposed method effectively improves the detection rate of SSD by 10.8% and thus provides guidance for evaluating the quality of optical material and optimizing optical material processing technologies. The research paradigm is equally applicable to biomedicine, energy, optoelectronics, and the environment, where QDs have a wide range of applications.

## 1. Introduction

The rapid development of large optical systems, such as lithography machines, astronomical telescopes, and high-resolution Earth observation systems, has imposed stringent requirements on the quality of optical materials. Owing to their brittle nature, almost all optical materials produce subsurface damage (SSD) during grinding, lapping, and polishing, which degrades their service life and laser damage threshold [1,2]. Therefore, SSD in optical materials must be accurately detected to enable the optimization of optical material processing technologies to eliminate SSD and thereby afford high-quality optical materials. However, SSD is difficult to detect because it is located below the optical material surface. Current SSD detection methods are either destructive or nondestructive in nature. The detection accuracy of destructive methods depends on the experience of the operator [3,4,5,6]. The effectiveness of nondestructive methods is limited by the surface roughness of components, so their SSD detection accuracy in ground and lapped optical material is low [7,8,9,10,11].

Since Brus proposed the concept of quantum dots (QDs) in 1983, they have been widely used in biomedical and optoelectronic devices [12,13,14]. In 2008, William et al. first proposed to use QDs to tag lapping slurries and detect SSD in lapped optical materials [15,16,17,18,19,20]. In 2018, Wang et al. used QDs to detect SSD depth and verified the effectiveness of this approach using the taper polishing method and magnetorheological polishing method [21]. In 2022, Cui et al. proposed detecting the SSD of optical materials by analyzing the photobleaching properties of QDs [22]. And Xiao et al. described a new method based on cadmium selenide/zinc sulfide (CdSe/ZnS) QDs that obtain quantitative three-dimensional information on SSD [23].

QDs are fluorescent nanomaterials that are sensitive to the polarization properties of excitation light [24,25]. However, existing QD-based SSD detection methods ignore the sensitivity of QDs to the polarization of excitation light, which means that their excitation efficiency and fluorescence collection efficiencies are low and, thus, SSD cannot be accurately detected.

To address these problems, this paper exploits the fluorescence polarization properties of QDs in the SSD of materials to improve the detection rate of SSD. First, the fluorescence polarization detection system for the SSD of optical materials was developed to collect fluorescence polarization images of QD-tagged optical materials under various excitation polarization angles and analyzer angles by investigating the detection principle of SSD in optical components based on the fluorescence polarization of QDs. And the fluorescence polarization properties of QDs in SSD were analyzed from excitation polarization and radiation polarization properties. Finally, the SSD of optical components was detected based on the fluorescence polarization properties studied. The results demonstrate that the detection rate of SSD can be improved by exploiting the fluorescence polarization properties of QDs. These findings can be used to evaluate the quality of optical components and optimize optical-component processing technologies to eliminate SSD.

## 2. Principles

QDs are fluorescent nanomaterials that exhibit photoluminescence. When subjected to excitation light, electrons outside the nucleus of fluorescent molecules absorb energy and move from their ground state to their excited state. The electrons in the excited state are highly energetic and unstable, and thus, return to their ground state by radiating their absorbed energy, thereby generating fluorescence. Fluorescent molecules excited by polarized light emit polarized light with anisotropy, which has different fluorescence intensities in different polarization directions. When a fluorescent molecule absorbs or emits photons, it can be regarded as an oscillating dipole with absorption and emission dipole moments. According to the principle of “light selection”, when linearly polarized light is used to excite a system with randomly oriented fluorescence molecules, the fluorescent molecules with absorption dipole moments parallel to the photon electric vectors, are preferentially excited, and the fluorescent molecule’s absorption rate of the excitation photon is proportional to cos^2^θ, where θ is the angle between the absorption dipole moments and the photon electric vectors [26,27]. This polarized emission depends on the (1) selective absorption of the excitation photons by the fluorescent molecule and (2) the orientation of the emitted photons. Therefore, the fluorescence polarization properties of QDs are reflected in the excitation polarization property, which depends on the absorption of fluorescent molecules, and the radiation polarization property, which depends on the fluorescent molecular anisotropy.

In order to improve the excitation and acquisition efficiency of the fluorescence of QDs in SSD, the excitation polarization properties of QDs in SSD were explored from the change in the fluorescence intensity of QDs in SSD with the polarization angle of linearly polarized excitation light; the radiation polarization properties of QDs in SSD were explored from the fluorescence anisotropy of QDs in SSD.

The fluorescence anisotropies of QDs in SSD were comprehensively characterized by horizontal anisotropy *R_H_*, vertical anisotropy *R_V_*, and integrated anisotropy *R*. Moreover, given the different sensitivities of detectors to vertically and horizontally polarized light in actual detection systems, the horizontal correction factor *G_H_*, vertical correction factor *G_V_*, and integrated correction factor *G* were considered. The polarization angle of the excitation light was adjusted using a polarizer, and the analyzer angle of the fluorescence was adjusted using an analyzer. The measured fluorescence intensity of the QDs in SSD is expressed as *I_XY_*. *X* and *Y* denote the orientations of the polarizer and analyzer, respectively. The orientations could be *H* or *V*, i.e., parallel or perpendicular, respectively. The fluorescence anisotropy of the SSD was calculated using Equations (1)–(6).

(1) $$R_{H}=\frac{I_{HH}-G_{H}I_{HV}}{I_{HH}+2G_{H}I_{HV}}$$

(2) $$G_{H}=\frac{I_{HV}}{I_{HH}}$$

(3) $$R_{V}=\frac{I_{VV}-G_{V}I_{VH}}{I_{VV}+2G_{V}I_{VH}}$$

(4) $$G_{V}=\frac{I_{VH}}{I_{VV}}$$

(5) $$R=\frac{I_{VV}-GI_{VH}}{I_{VV}+2GI_{VH}}$$

(6) $$G=\frac{I_{HV}}{I_{HH}}$$

Owing to the two-dimensional nature of the transition dipole of QDs, the sign of fluorescence anisotropy in QDs with SSD may change when the excitation polarization changes from the vertical position to the horizontal position [28], i.e., the positive and negative signs of the vertical and horizontal anisotropies may not coincide. In this study, the focus was on the magnitude of anisotropy instead of its sign, and thus, only the magnitudes of anisotropy were compared. In addition, photobleaching must be minimized as it decreases the number of fluorophores, thereby decreasing the accuracy of fluorescence measurements.

**Figure 1:**
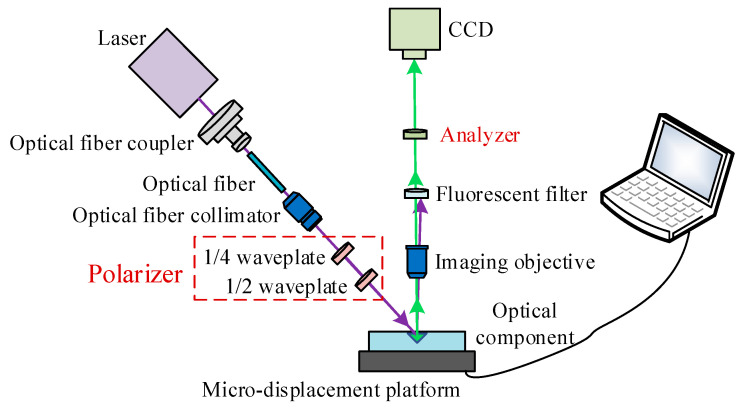
Schematic diagram of the fluorescence polarization detection system for the SSD of optical materials.

Based on the above analysis, the schematic diagram of the fluorescence polarization detection system for the SSD of optical components was designed, as shown in Figure 1. In order to improve the detection accuracy of SSD in optical components, this paper further considers the polarization sensitivity of QDs to the excitation light on the basis of the traditional method, and adopts the waveplates as a polarizer, so as to make adjustments to the polarization state of excitation light without changing the excitation light power.

The fiber-coupled laser emitted a randomly polarized laser, which was adjusted by a polarizer to yield a linearly polarized laser beam. The linearly polarized laser excited the QDs in the SSD of the optical materials to emit fluorescence, and the fluorescence was separated from the laser by a fluorescence filter after it passed through the imaging objective. The laser beam was reflected back, whereas the fluorescence passed through the fluorescence filter to reach the analyzer and was imaged by the charge-coupled device (CCD). A linearly polarized laser beam was obtained by adjusting the 1/4 waveplate, and the 1/2 waveplate was adjusted to obtain different excitation polarization angles.

## 3. Experiments

### 3.1. QD-Tagged SSD in Optical Materials

The fused silica optical material was taken as the research object in this paper. Previous studies demonstrated that the detection of SSD in fused silica can be realized based on the green autofluorescence at the peak position of 550 nm [29]. However, its autofluorescence was too weak, which was not conducive to the accurate detection of SSD. Therefore, it was necessary to use QDs to tag the SSD to enhance its fluorescence intensity. The samples were fused silica optical materials with dimensions of Φ25 × 3 mm. Before being used for this study, the samples were polished sufficiently to remove the SSD generated by grinding and lapping to prevent it from influencing the results.

According to the composition of the core elements of QDs, QDs can be categorized into unitary QDs, binary QDs, ternary QDs, and so on. Unitary quantum dots are mainly group IV QDs, such as carbon QDs (C-QDs) and silicon QDs (Si QDs). Binary QDs are mainly group II–VI and III–V QDs, which can be divided into mononuclear QDs and core/shell QDs, such as cadmium selenide QDs (CdSe QDs), cadmium selenide/zinc sulfide QDs (CdSe/ZnS QDs), indium phosphide QDs (InP QDs), and indium phosphide/zinc sulfide QDs (InP/ZnS QDs). Ternary QDs mainly belong to group I-III-VI QDs, such as copper indium sulfide/zinc sulfide QDs (CuInS_2_/ZnS QDs). Based on our previous studies [22], the properties of the various QDs mentioned above were fully compared, and the results of the study showed that the stability and anti-photobleaching properties of C-QDs were stronger. So, in this paper, C-QDs were used to tag SSD in optical materials. In addition, the QDs used in this paper were purchased from Xingzi New Material Technology Development Co., Ltd. (Shanghai, China), and their model was WB11154-10.

**Figure 2:**
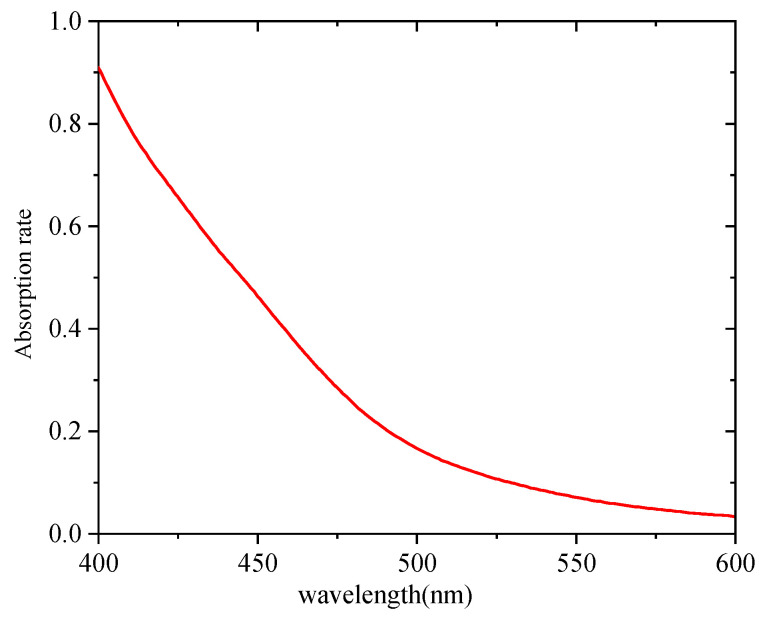
Absorption spectrum of C-QDs.

**Figure 3:**
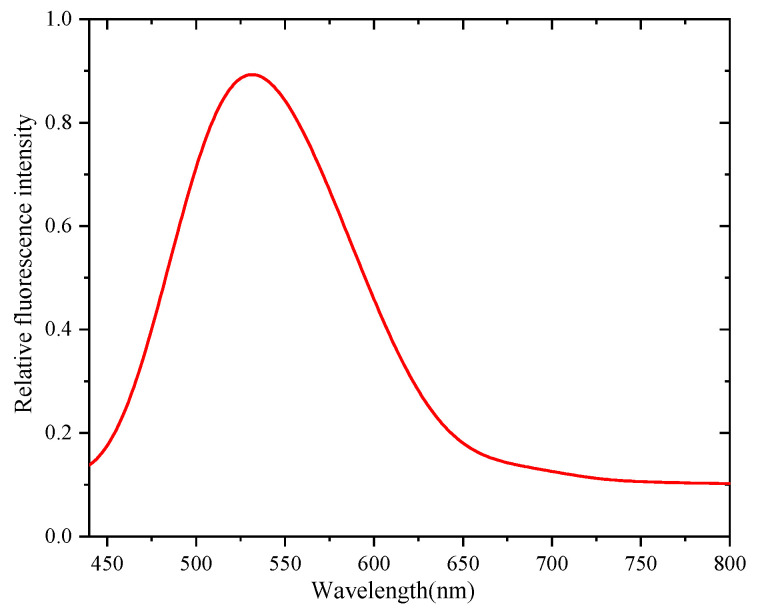
Fluorescence spectrum of C-QDs.

The absorption and fluorescence spectra of the C-QDs used herein were obtained using a UV-Vis spectrophotometer and a fluorescence spectrophotometer, respectively. The results are shown in Figure 2 and Figure 3, respectively.

As shown in Figure 2 and Figure 3, the C-QDs could be excited by short-wavelength light, and the fluorescence peak of the C-QDs was at 544 nm. According to previous related research, optical materials were processed using QD-labeled abrasive solutions, enabling the QDs to enter the subsurface layer of the optical material with the lapping process, realizing the tagging of SSD [15,16,17,18,19,20,21]. The detailed processing steps and parameters are shown in Table 1. The QDs used in the experiments were water soluble, and the dose was 10 mg.

### 3.2. Fluorescence Polarization Detection System for the SSD of Optical Materials

**Figure 4:**
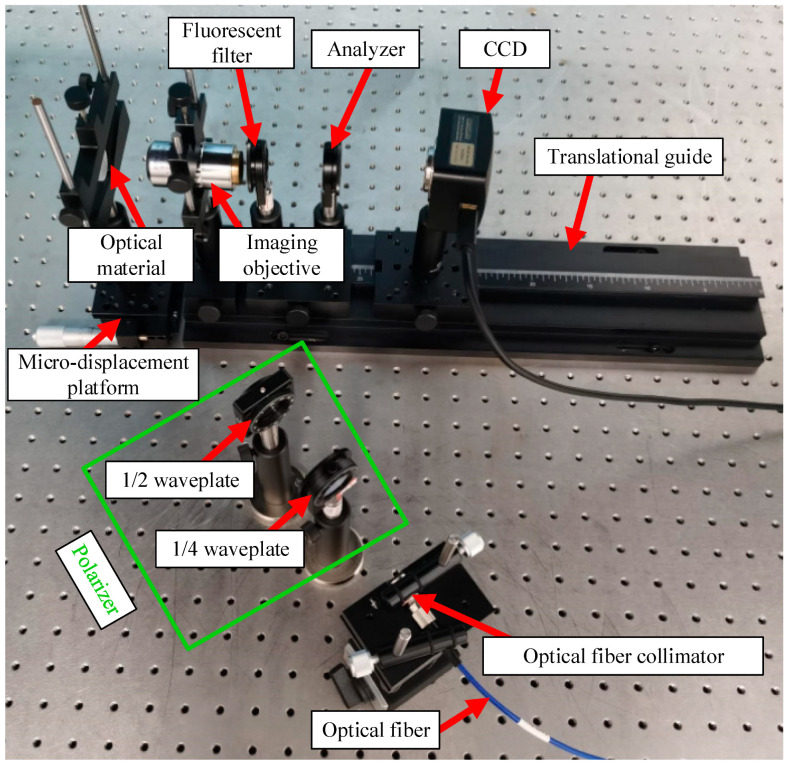
Fluorescence polarization detection system for the SSD of optical materials.

The developed fluorescence polarization detection system for the SSD of optical materials is shown in Figure 4. Because QDs could be excited by short-wavelength light, a fiber-coupled laser with a wavelength of 405 nm was used as the excitation source. The laser emitted from the fiber-coupled laser was elliptically polarized, as tested by a polarization state meter. Table 2 lists the key parameters of the instruments constituting the system.

The fluorescence polarization detection system for the SSD of optical materials was used to collect fluorescence polarization images of SSD in the optical materials tagged by C-QDs under various excitation polarization angles and analyzer angles. The polarization state was measured by a polarization state meter when adjusting the excitation angle to ensure the reliability of the experimental results.

## 4. Results and Discussion

### 4.1. Characterization of Fluorescence Polarization Properties of the QDs in SSD

The fluorescence polarization properties of C-QDs in SSD were analyzed from fluorescence excitation polarization and radiation polarization properties.

#### 4.1.1. Characterization of Excitation Polarization Properties

An SSD was arbitrarily selected on the C QD-tagged optical material, and its fluorescence images were collected according to the following steps:

(1) Remove the 1/4 waveplate, 1/2 waveplate, and analyzer in the system shown in Figure 4, keep other devices unchanged, and collect the initial fluorescence image J_0_ of the SSD without considering the fluorescence polarization characteristics, and extract its initial fluorescence intensity I_0_ as the initial reference value. At this time, the elliptically polarized light generated by the laser is directly applied to the fused silica optical materials without an analyzer.
(2) Remove only the analyzer in the system shown in Figure 4, leaving the other devices unchanged, and adjust the excitation light (elliptically polarized light) to linearly polarized light using a 1/4 waveplate, and then adjust the 1/2 waveplate to obtain different polarization angles. The polarization angle of the linearly polarized light was adjusted from 0° to 90°, and the fluorescence polarization image of the SSD was acquired every 10°. The fluorescence polarization images acquired at excitation polarization angles of 0°, 10°, 20°, …, 90° were noted as J_10_, J_11_, J_12_, …, J_19_, and the corresponding fluorescence intensities I_10_, I_11_, I_12_, …, I_19_ were extracted, respectively.

**Figure 5:**
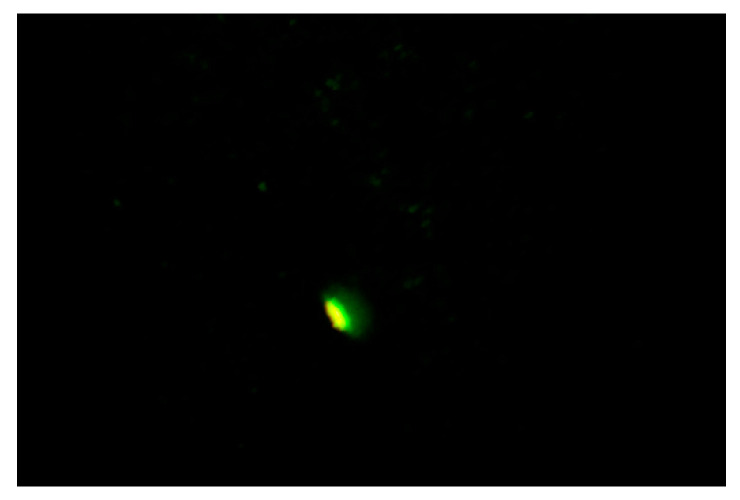
The initial fluorescence image J_0_ of the SSD tagged by C-QDs.

**Figure 6:**
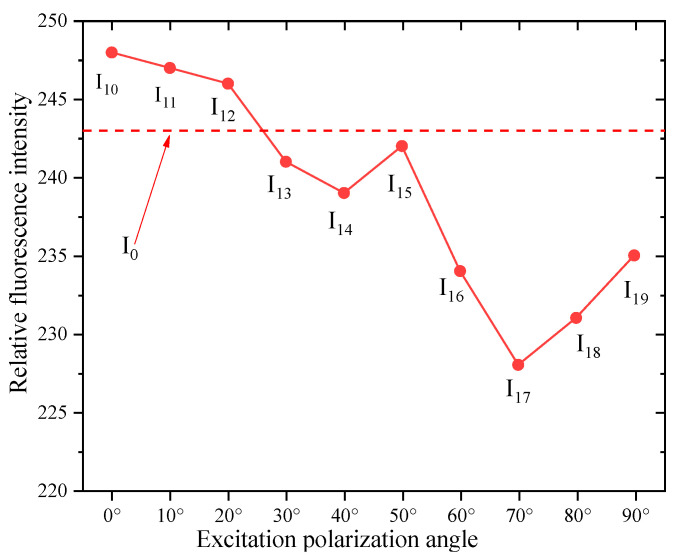
The variation in fluorescence intensity of the SSD with excitation polarization angle.

The acquired initial fluorescence image J_0_ is shown in Figure 5. The fluorescence intensity of the SSD is characterized using the gray value of the fluorescence image. Then, the variation in fluorescence intensity of the SSD with excitation polarization angle is shown in Figure 6.

During the experiment, the fluorescence images were acquired in the sequence of J_0_, J_10_, J_11_, J_12_, …, J_19_. Therefore, the later the fluorescence images were acquired, the longer the excitation time and the more the corresponding fluorescence intensity decayed, i.e., I_0_, I_10_, I_11_, I_12_, …, I_19_ decreased accordingly. From Figure 6, it can be seen that I_0_, I_10_, I_11_, I_12_, …, I_19_ did not decrease accordingly. The fluorescence intensity of C-QDs in the SSD of the optical materials is greater than its initial fluorescence intensity when the excitation polarization angle is 0° to 20°. The fluorescence intensity of C-QDs in the SSD of the optical components is less than their initial fluorescence intensity when the excitation polarization angle is 30° to 90°, and this fluorescence attenuation may be due to the long duration of image acquisition and excitation. The fluorescence intensities at excitation polarization angles of 50°, 80°, and 90° all increased relatively from their previous excitation polarization angles (i.e., 40°, 70°, and 80°), suggesting that the polarization angle of the excitation light affects the fluorescence intensities of QDs in SSD.

Given the time required for data acquisition when laser beams with different excitation polarization angles act on the same SSD, fluorescence attenuation occurs at this SSD, which affects the detection results. Therefore, laser beams with different excitation polarization angles were applied to different types of SSD, respectively. To characterize the excitation polarization properties of the QDs in SSD, the effect of the excitation polarization angle on the fluorescence intensity was quantitatively investigated by the fluorescence intensity change rate *γ*.

The excitation polarization angle varied from 0° to 90° in intervals of 10°. Thus, 10 positions were arbitrarily selected on the components tagged by C-QDs. On each position, fluorescence polarization images J_1_′ and initial images J_0_′ (without considering the fluorescence polarization characteristics) were collected, and their corresponding fluorescence intensities *I*_1_′ and *I*_0_′ were extracted. To acquire J_1_′ using the system in Figure 4, the analyzer was removed, and the polarizer was adjusted to obtain a linearly polarized laser beam with specified excitation polarization angles. To acquire J_0_′ using the system in Figure 4, the polarizer and analyzer were removed. *γ* was calculated using Equation (7).

(7) $$\gamma =\frac{I_{1}^{\prime }-I_{0}^{\prime }}{I_{0}^{\prime }}$$

Positive and negative *γ* mean that the polarized light enhanced and suppressed the fluorescence intensity of the SSD, respectively. The optimal polarization excitation angle Ex was the angle that corresponded to the maximum *γ*.

**Figure 7:**
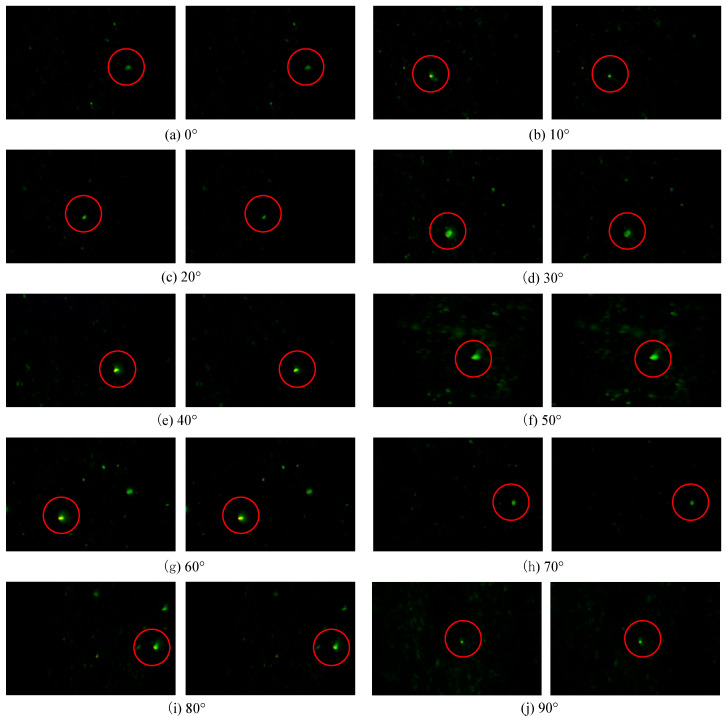
(**a**–**j**) The fluorescence initial images J_0_′ (the left) and fluorescence polarization images J_1_′ (the right) of SSD at different locations on the optical materials tagged with C-QDs.

**Figure 8:**
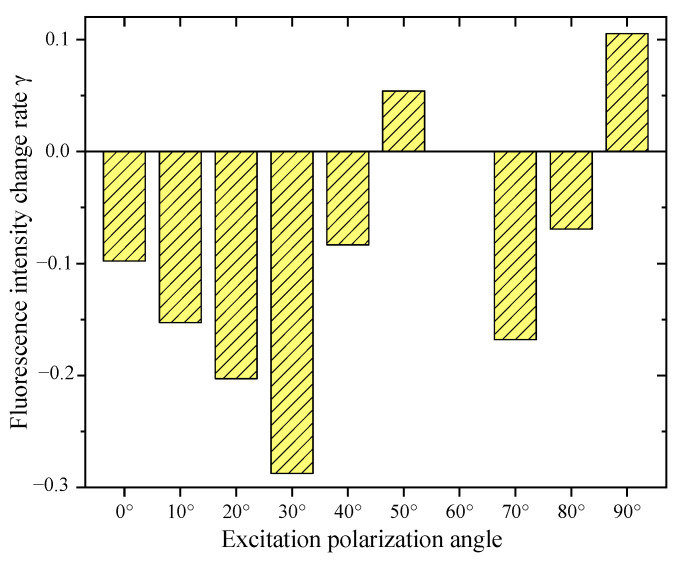
The γ at different excitation polarization angles.

The fluorescence initial images J_0_′ (the left) and fluorescence polarization images J_1_′ (the right) of SSD at different locations on the optical components tagged with C-QDs are shown in Figure 7. In Figure 6, multiple SSDs were detected at most locations, and for ease of analysis, the SSDs with high fluorescence intensity were selected for the study and marked out using red circles. Figure 8 shows the *γ* at different excitation polarization angles.

From Figure 8, the following results can be determined. (1) The fluorescence of C-QDs in SSD was not consistent in its response to the excitation polarization angle. In general, the excitation light with different excitation polarization angles inhibited or enhanced the fluorescence of SSD, but at a certain polarization angle, it did not affect the fluorescence. The reason why the fluorescence intensity of QDs was unchanged at a certain excitation polarization angle was that the angle θ between the excitation photon vector and the dipole moment of the QD absorption was unchanged before and after the action of the excitation polarized light. Therefore, the overall absorption rate of the QDs of the SSD for the excitation light was unchanged, which led to no change in the final fluorescence intensity. (2) The *γ* of the C-QDs in the SSD was the largest at the excitation polarization angle of 90°. Therefore, the Ex of the C-QDs in the SSD was 90°.

This analysis indicated that in order to improve fluorescence excitation efficiency, the SSD of the optical materials tagged by C-QDs should be excited by linearly polarized light with polarization angles of 90°.

In addition, due to the differences in the structure of the SSDs, the randomness of the distribution of QDs in the SSD, and the inconsistency of the orientation of the electric dipole moments of the QDs, the angle between the absorption dipole moment of each QD in SSD and the excitation light are not exactly the same, resulting in a difference in fluorescence intensity produced by each QD. Thus, the fluorescence intensity of the laser with the same polarization state acts on different SSDs or the fluorescence intensity of the laser with different polarization states acts on the same SSD differently. To address this problem, this study involved conducting many repeated experiments, which show that the optimal excitation angle is not always 90°, but, in general, is concentrated in the range of 50° to 90°.

#### 4.1.2. Characterization of Radiation Polarization Properties

The fluorescence radiated by QDs under the action of excitation light is anisotropic, and thus, different fluorescence intensities can be detected in different polarization directions using an analyzer. Therefore, the radiative polarization characteristics of SSD in optical materials were investigated from the fluorescence anisotropy of SSD.

Six positions (P1, P2, …, P6) were arbitrarily selected on the fused silica optical materials tagged with C-QDs. And the fluorescence anisotropy of the SSD was calculated as described in Section 2. The results are summarized in Table 3.

Table 3 shows that (1) although the fluorescence anisotropy of the QDs in SSD is very low, they are anisotropic in both the horizontal and vertical directions, which indicates that the fluorescence of SSD can be detected in all polarization directions and the fluorescence is elliptically polarized; (2) the vertical anisotropy is stronger than the horizontal anisotropy for most of the SSDs (P1, P2, P3, P5, P6), and the horizontal anisotropy is stronger than the vertical anisotropy for only a few individual types of SSD (P4), which further proves that the orientation of the fluorescent molecules themselves is preferred at 50–90° for most of the QDs in the SSDs. When the excitation light with a polarization angle of 50–90° acts on the QDs in the SSDs, the angle θ between the absorption dipole moments of the fluorescent molecules of the QDs and the photon electric vectors is closer to 0. Since the fluorescent molecule’s absorption rate of the excitation photon is proportional to cos^2^θ, the excitation efficiency of QDs is highest when θ is close to 0. Therefore, the excitation efficiency of QDs can be significantly improved using excitation light with a polarization angle of 50–90°.

The above analysis indicated that in order to improve the detection accuracy of SSD, the SSD of the tagged optical materials could be excited by linearly polarized light with a polarization angle of 50–90°.

### 4.2. The Detection of SSD in the Optical Materials Based on the Fluorescence Polarization Properties of QDs

To demonstrate the accuracy of detecting the SSD based on the fluorescence polarization properties of QDs, the analyzer in the fluorescence polarization detection system for the SSD of optical components was removed, and the fluorescence polarization images of the SSD in the tagged components were acquired under the excitation of linearly polarized light with optimal polarization angles. Although the range of the optimal polarization angle was 50–90°, linearly polarized light at 90° is often used as the reference direction in the optical system, which is more stable. However, the angle within [50, 90) needs to be controlled by the angle scale or software, which is susceptible to mechanical errors. Therefore, 90° was adopted here as the optimal polarization angle.

**Figure 9:**
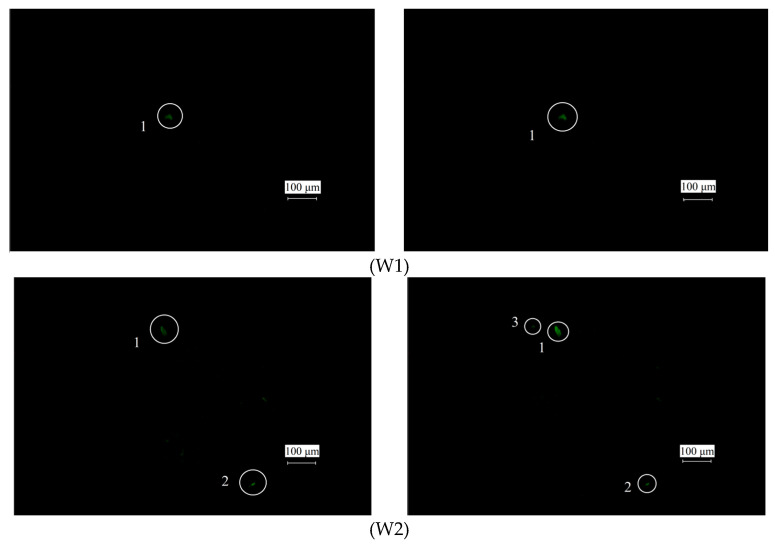

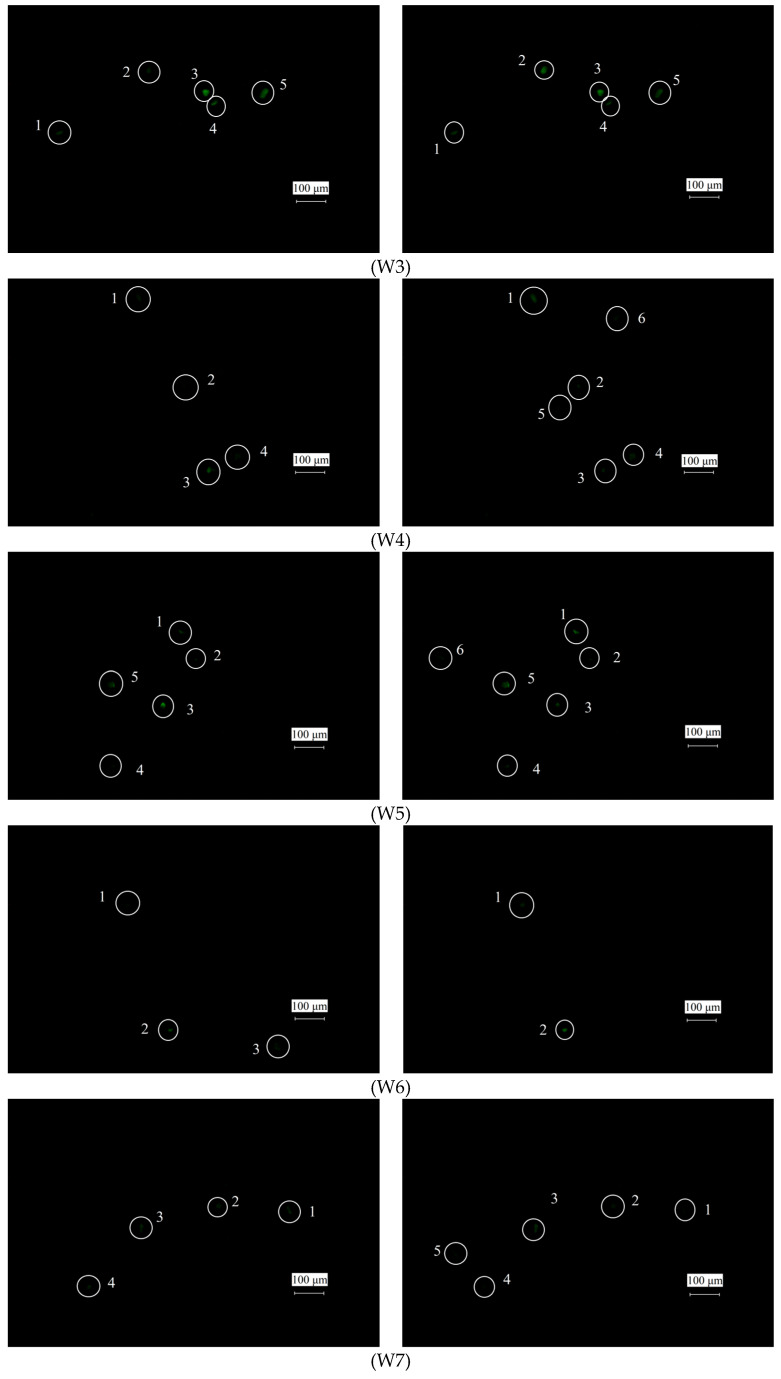

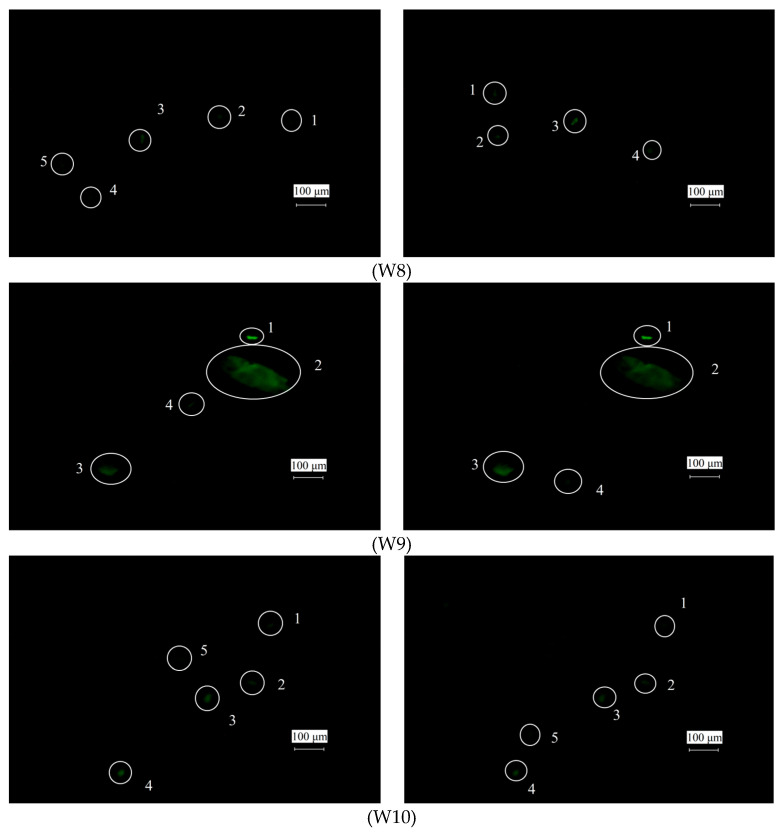
The initial fluorescence images (**left**) and fluorescence polarization images (**right**) of SSD in tagged optical materials.

The fluorescence polarization images were acquired at 10 arbitrarily selected positions (W1–W10) on the tagged materials. The initial fluorescence images of the SSD at the 10 positions were also acquired as a control without considering the fluorescence polarization characteristics (i.e., elliptically polarized light generated by the laser directly excites the components, and there is no analyzer). The fluorescence polarization images and fluorescence initial images of SSD are shown in Figure 9, where the left is the fluorescence initial image and the right is the fluorescence polarization image.

Table 4 summarizes the number of SSD sites detected in the cases shown in Figure 9, and it indicates that the amount of SSD detected after considering the polarization increased by 10.8%, which indicates that the detection rate of SSD increased by 10.8% when detecting SSD based on the fluorescence polarization characteristics of QDs.

## 5. Conclusions

This paper describes a novel method for detecting the SSD in optical materials by exploiting the fluorescence polarization properties of QDs. The studies show that in order to maximize the fluorescence excitation and collection efficiencies, the SSD of the optical components tagged by C-QDs can be excited by linearly polarized light with polarization angles of 50–90°. And compared with the SSD detected without considering the polarization, the amount of SSD detected after considering the polarization increased by 10.8%, which indicates that the detection rate of SSD can be increased by exploiting the fluorescence polarization properties of QDs. This study can provide guidance for evaluating the quality of optical materials and optimizing optical-component processing technologies to remove SSD. This research is of great significance for the development of large-scale optical systems. And the research paradigm is equally applicable to biomedicine, energy, optoelectronics, and the environment, where QDs have a wide range of applications in order to address real-world problems in related fields.

## Figures and Tables

**Table 1:** Optical material processing steps and parameters.

Steps	Processing Steps and Parameters
1	D30 water-based diamond abrasive; lapping time = 5 min; lapping speed = 54 rad/min
2	D6 water-based diamond abrasive; lapping time = 3 min; lapping speed = 54 rad/min
3	Ultrasonically cleaned using an ethanol solution; cleaning time = 5 min. (Adequately removing impurities and residual quantum dots from the surface of optical materials)

**Table 2:** The key parameters of the instruments.

Number	Instruments	Key Parameters
1	Fiber-coupled laser	Wavelength: 405 nm; Output power: 50 mw.
2	1/4 waveplate	Wavelength: 405 nm; Delay accuracy: λ/300.
3	1/2 waveplate	Wavelength: 405 nm; Delay accuracy: λ/300.
4	Imaging objective	Magnification: 10×; Numerical aperture: 0.28; Working distance: 33.4 mm; Focal length: 200 mm.
5	Fluorescence filter	Center wavelength: 544 nm; Transmissivity: >96%; Cut off depth: OD6.
6	Analyzer	Wavelength range: 475–625 nm; Transmissivity: >55–81%; Extinction ratio: >1000:1.
7	CCD	Sensor model: IMX178; Spectral response: 400~1100 nm.

**Table 3:** Fluorescence anisotropy of SSD in optical materials.

Parameters		Positions					
		P1	P2	P3	P4	P5	P6
Fluorescence intensity	*I_HV_*	67	136	74	119	84	87
	*I_HH_*	66	132	73	125	83	87
	*I_VH_*	62	146	74	119	66	82
	*I_VV_*	64	151	84	121	73	88
Correction factor	*G_H_*	1.015	1.030	1.014	0.952	1.012	1
	*G_V_*	0.969	0.967	0.881	0.983	0.904	0.932
	*G*	1.015	1.030	1.014	0.952	1.012	1
Anisotropy	*R_H_*	−0.010	−0.020	−0.009	0.033	−0.008	0
	*R_V_*	0.021	0.023	0.088	0.011	0.069	0.048
	*R*	0.006	0.001	0.038	0.022	0.030	0.024

**Table 4:** The amount of SSD in Figure 9.

Positions	Fluorescence Initial Image	Fluorescence Polarization Images
W1	1	1
W2	2	3
W3	5	5
W4	4	6
W5	5	6
W6	3	2
W7	4	5
W8	4	4
W9	4	4
W10	5	5
Sum	37	41

## Data Availability

The data underlying the results presented in this paper are not publicly available at this time but may be obtained from the authors upon reasonable request.
